# Clenching the Strings of Bruxism Etiopathogenesis: Association Analyses on Genetics and Environmental Risk Factors in a Deeply Characterized Italian Cohort

**DOI:** 10.3390/biomedicines12020304

**Published:** 2024-01-28

**Authors:** Alessandro Pecori, Valentina Luppieri, Aurora Santin, Beatrice Spedicati, Stefania Zampieri, Milena Cadenaro, Giorgia Girotto, Maria Pina Concas

**Affiliations:** 1 Institute for Maternal and Child Health—IRCCS “Burlo Garofolo”, Via dell’Istria 65, 34137 Trieste, Italy; alessandro.pecori@burlo.trieste.it (A.P.); valentina.luppieri@burlo.trieste.it (V.L.); beatrice.spedicati@burlo.trieste.it (B.S.); stefania.zampieri@burlo.trieste.it (S.Z.); mcadenaro@units.it (M.C.); giorgia.girotto@burlo.trieste.it (G.G.); mariapina.concas@burlo.trieste.it (M.P.C.); 2 Department of Medicine, Surgery and Health Sciences, University of Trieste, Strada di Fiume, 447, 34149 Trieste, Italy

**Keywords:** bruxism, etiology, epidemiologic study, genome-wide association study, anxiety disorders, isolated populations

## Abstract

Bruxism is a worldwide oral health problem. Although there is a consensus about its multifactorial nature, its precise etiopathogenetic mechanisms are unclear. This study, taking advantage of a deeply characterized cohort of 769 individuals (aged 6–89 years) coming from Northern Italy’s genetically isolated populations, aims to epidemiologically describe environmental risk factors for bruxism development and identify genes potentially involved through a Genome-Wide Association Study (GWAS) approach. Logistic mixed models adjusted for age and sex were performed to evaluate associations between bruxism and possible risk factors, e.g., anxiety, smoking, and alcohol and caffeine intake. A case-control GWAS (135 cases, 523 controls), adjusted for age, sex, and anxiety, was conducted to identify new candidate genes. The GTEx data analysis was performed to evaluate the identified gene expression in human body tissues. Statistical analyses determined anxiety as a bruxism risk factor (OR = 2.54; 95% CI: 1.20–5.38; *p*-value = 0.015), and GWAS highlighted three novel genes potentially associated with bruxism: *NLGN1* (topSNP = rs2046718; *p*-value = 2.63 × 10^−7^), *RIMBP2* (topSNP = rs571497947; *p*-value = 4.68 × 10^−7^), and *LHFP* (topSNP = rs2324342; *p*-value = 7.47 × 10^−6^). The GTEx data analysis showed their expression in brain tissues. Overall, this work provided a deeper understanding of bruxism etiopathogenesis with the long-term perspective of developing personalized therapeutic approaches for improving affected individuals’ quality of life.

## 1. Introduction

An international consensus, published in 2013, described bruxism as a “repetitive jaw-muscle activity characterized by clenching or grinding of the teeth and/or by bracing or thrusting of the mandible with two distinct circadian manifestations: it can occur during sleep, indicated as sleep bruxism (SB), or during wakefulness, indicated as awake bruxism (AB)” [1].

By increasing the workload on the stomatognathic system, bruxism may determine negative consequences at different compartments: (i) at the teeth level, i.e., mechanical teeth wear, cracks, recurring failure of conservative and/or prosthetic restorations due to dental attrition and dentin hypersensitivity [2,3], and (ii) at the muscular and articular level, causing hypertrophy and pain of masticatory muscles, hypertonicity and pain of cervical muscles, tension-type headache, and temporomandibular joint (TMJ) disorders, including click, noise, and pain [2,3,4,5,6].

The reported prevalence of bruxism is highly variable; this wide variability might reflect different characteristics of the studied populations, such as age and sex distribution and socioeconomic status, as well as methodological issues in data collection, including a lack of standardized diagnostic criteria application [3]. A systematic review published in 2013 reports a prevalence rate of bruxism in adults ranging from 9.3% to 15.3% for SB and from 22.1% to 31% for AB [3,4,5,6,7]. In children and adolescents, the prevalence rates increase up to 40–50% [3,4,5,6,8].

Although the specific pathogenetic mechanisms underlying the etiology of bruxism are still controversial and poorly elucidated, there is a consensus about its multifactorial origin [3,9,10,11], meaning that its onset is determined by a complex interaction of genetics and environmental factors. Regarding the environmental aspect, to date, several possible causes have been described, such as morphological (i.e., interferences in dental occlusion and articulation [10]), pathophysiological (i.e., smoking, alcohol, caffeine consumption, sleep disturbances, certain medications pronged intake, drug abuse [10,12,13]), and psychosocial factors (i.e., anxiety, depression, stress, mood disorders [10,14,15,16]). 

However, it has been considered that the role of morphological factors in bruxism onset has been resized recently and is considered marginal; conversely, an increased focus has been placed on the psychosocial and pathophysiological factors that support bruxism as a CNS-mediated phenomenon [10]. Different pathological mechanisms related to the central nervous system (CNS), such as sleep-related dysfunctions and imbalanced neurotransmission, are in fact assumed to cause bruxism [17,18].

Concerning the genetics of bruxism, the molecular mechanisms underlying its etiology are still poorly characterized. To date, most of the published studies are focused on functional validations of a few possible candidate genes, and, as far as it is known, only one Genome-Wide Association Study (GWAS) on SB is available in the literature [19].

Overall, considering the current literature picture on the genetics of bruxism, the genes described so far can be grouped into two main categories: (i) brain-related genes, such as metalloproteinase genes (*MMP2*, *MMP9*), catechol-o-methyltransferase genes (*COMT*) [20], dopaminergic genes (*DRD1*, *DRD2*, *DRD3*, *DRD4*, *DRD5*) [21,22], serotonin receptor genes (*5-HT1A*, *5-HT2A*) [23], and genes involved in stress regulation (*NTRK2*, *BDNF*) [24], and (ii) genes related to muscle activity regulation, such as *ACTN* [25] and *MYO3B* [19] genes. 

However, despite the efforts made so far, a limited understanding of bruxism genetics has been reached.

To study multifactorial traits and disorders, Genome-Wide Association Studies (GWAS) on isolated populations have been considered a successful strategy for detecting novel candidate genes [26]. Indeed, isolated populations, being characterized by high genetic and environmental homogeneity, can facilitate the identification of traits and disease etiological factors since the variance of the genetic and environmental background is minimal [27,28].

Therefore, in order to shed light on the knowledge underlying bruxism etiopathogenesis both at the environmental and genetic levels, the present study aims to take advantage of a deeply clinically and genetically characterized cohort of isolated populations in North-Eastern Italy, within the context of the “Friuli-Venezia Giulia (FVG) Genetic Park” project [29]. The “FVG Genetic Park” belongs to the so-called project “Italian Network of Genetic Isolates (INGI)”, which is a cooperation between several research Italian institutions; the final goal of this collaboration is the identification of genes, variants, and environmental factors involved in multifactorial traits and disease etiopathogenesis [30].

In detail, the purposes of this study are to: (1) epidemiologically characterize bruxism and potential risk factors in an Italian genetically isolated cohort; and (2) identify new genes potentially associated with bruxism through a GWAS analysis approach.

## 2. Materials and Methods

### 2.1. Study Design and Ethical Committee Approval

This study was designed as an epidemiological cross-sectional study performed on data collected between March and November 2008 in five villages of the FVG region, Italy. 

Further, it was approved by the Ethical Committee of the Institute for Maternal and Child Health—IRCCS “Burlo Garofolo”, Trieste, with the univocal code Prot. CE/V-78, 06/08/2007. 

A written informed consent to the study was given to all participants; for underage individuals, the informed consent was signed by parents or legal guardians.

This study was conducted according to the ethical principles of the Helsinki Declaration.

### 2.2. Study Population

Here, 769 subjects coming from isolated FVG region villages (i.e., Erto-Casso, Clauzetto, Illegio, Sauris, and Val di Resia) were enrolled, voluntarily, between March and November 2008 in the odontostomatological evaluation within the context of the “FVG Genetic Park” project [6]. 

The considered villages met the criteria for being defined as “genetic isolates”; specifically, they are located in distinct geographical sites, and since they have originated from a founder event [27,28], they are characterized by few founder individuals, high endogamy rates, language barriers, low variability of surnames, and very rare events of emigration and immigration. 

Inhabitants were invited to participate in the research program by public advertisements through different communication channels, such as local authorities and physicians, spots on television, newspaper articles, and mailing; several meetings between researchers and the population were also organized to present the project.

In this study, individuals presenting primary dentition and edentulous were discarded from the statistical analysis. For GWAS analysis, only individuals with available genetic data were included (n = 658).

### 2.3. Demographic and Clinical Data Collection

A structured questionnaire (>200 questions) was administered to collect demographic data (e.g., sex, age, and village of origin) and lifestyle habit information (e.g., smoking, caffeine, and alcohol consumption). Further, an accurate personal and familiar anamnesis was collected from each participant. Each enrolled subject underwent detailed evaluations, e.g., cardiovascular, neurological, in-depth sensorial, and odontostomatological assessments [5]. Biochemical and metabolomic parameters were also analyzed. All these data were systematically annotated by professionals and registered according to a standardized format. Diseases were classified according to the International Classification of Diseases and Related Health Problems—10th Revision (ICD-10) [31].

Bruxism was evaluated by a calibrated dentist and clinically diagnosed considering both the presence of signs of tooth wear (due to attrition) on opposing teeth, i.e., on anterior permanent teeth incisal surfaces and/or posterior permanent teeth guiding cusps, pain at the palpation of the masticatory muscles (temporalis and masseter), and/or hypertrophy of these muscles evaluated at palpation and visual inspection [1,2,3]. In addition, participants were asked if they were aware of tooth grinding and/or clenching.

### 2.4. DNA Extraction, Genotyping, and Imputation

DNA was extracted from blood samples. Genotyping was performed with an Illumina 370k/700k/MEGA SNP array (Illumina Inc., San Diego, CA, USA). Genotype calling was conducted using Illumina GenomeStudio. The following standard quality control criteria were applied: sample call rate ≥ 0.95, gender check, SNP call rate ≥ 0.95, Hardy–Weinberg Equilibrium *p*-value > 1 × 10^−^^6^, and minor allele frequency (MAF) ≥ 0.01. Genotype data were then imputed with IMPUTE2 version 2.3.2 [32], employing as a reference a customer panel generated by the 1000 Genomes phase 3 [33] and INGI samples of whole-genome sequences [30]. Afterward, a single Variant Call Format file was generated; and the Info score and allele frequencies were estimated with QCTOOL software version 2. SNPs with MAF < 0.05 and Info score < 0.4 were excluded from the analyses.

### 2.5. Statistical Analyses

A descriptive statistical analysis was carried out; categorical variables were described with numbers and percentages, while continuous variables, normally distributed, were described with mean and standard deviation and, otherwise, with median and interquartile range.

Associations between bruxism and the following possible risk factors were tested: anxiety disorder (code F 41.9 of the ICD-10), depressive disorder (code F 32.2 or F 32.9 of the ICD-10) [31], smoking habit (yes/no), number of cigarettes/day, caffeine consumption (yes/no), number of cups/day, alcohol consumption (yes/no), and alcohol grams/day. Bruxism and depressive/anxiety disorders were registered as the presence (value 1) or absence (value 0) of the disease/condition; smoking habits were coded 1 for current smokers and 0 for current non-smokers; alcohol consumption as 1 for current drinkers and 0 for current non-drinkers; and finally, caffeine consumption was classified as 1 for current coffee drinkers and 0 for current non-coffee drinkers. The Chi-square test was performed to analyze possible associations between bruxism and each possible categorical risk factor, while the *t*-test, or, if not normally distributed, the Mann–Whitney U test, was performed to assess associations between bruxism and each possible continuous risk factor.

Thereafter, any variable significantly associated with bruxism was tested using logistic mixed-effect regression models to verify whether it could be an independent predictor of the outcome (bruxism). The logistic mixed effect models were implemented using the lme4 package version 1.1-34 in R software version 4.1.2 (R Foundation for Statistical Computing, Vienna, Austria). Age (a continuous variable), sex, and village of origin were included as covariates, specifically the first two as fixed effects and the third as a random effect.

The statistical significance threshold was fixed at a *p*-value < 0.05.

The post hoc power calculation for the logistic model (WebPower package version 0.9.4 of the R software version 4.1.2) on the relationship between bruxism and anxiety disorder, considering the probability of having a positive outcome (Y = 1) in the presence of the risk factor (X = 1) of 0.4062, the probability of having a positive outcome (Y = 1) in the absence of the risk factor (X = 0) of 0.201, and an alpha of 0.05, has a statistical power of 0.999.

### 2.6. GWAS Analysis

A logistic regression was performed for GWAS, assuming an additive genetic model corrected for anxiety, sex, age, village of origin, and the first ten principal components, using the REGENIE software version 3.3.3 [34]. SNPs with a *p*-value < 5 × 10^−^^8^ were considered genome-wide significant, while SNPs with a *p*-value < 1 × 10^−^^5^ as suggestive. All SNPs with a *p*-value < 1 × 10^−^^5^ were annotated with the Variant Effect Predictor tool (VEP, https://www.ensembl.org/info/docs/tools/vep/index.html, accessed on 5 July 2023) [35]. VEP allows to identify the SNPs closest genes and derive the related functional features. Only protein-coding genes were considered, while long non-coding RNA (LINC) genes, genes with unknown functions named with LOC and FAM symbols, or pseudogenes were discarded from the analysis. Only the genomic *loci* with at least five SNPs in linkage disequilibrium (LD) and with *p*-value < 1 × 10^−5^ in around 250 kb were considered. Data were aligned to the Human genome reference build 37 (GRCh37).

### 2.7. Genotype-Tissue Expression (GTEx) Database Analysis

Expression levels of the identified genes by GWAS analysis were checked into GTEx dataset release v8 [36], focusing on the known tissues involved in bruxism manifestation, such as brain and skeletal muscle tissues [17].

**Figure 1:**
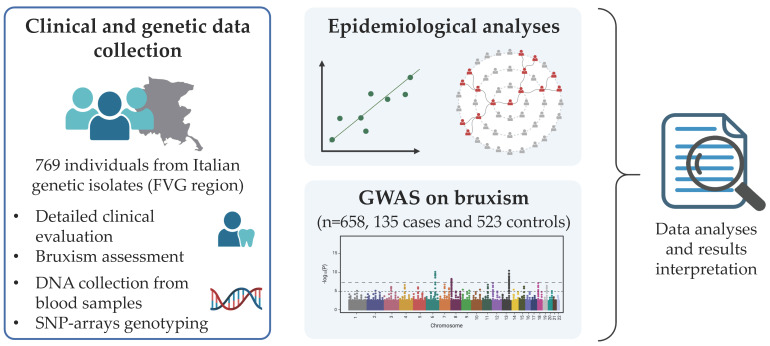
Study workflow. This figure summarizes the study workflow. In particular, 769 individuals from an Italian genetic isolate (FVG region) underwent a detailed clinical evaluation, with a specific focus on bruxism assessment. DNA was extracted from blood samples and genotyped. Epidemiological analyses were performed on the total sample to evaluate the prevalence of bruxism and possible risk factors. A GWAS analysis on bruxism was conducted on a cohort of 658 individuals (135 cases and 523 controls). The results were then analyzed and interpreted in relation to the literature.

The complete workflow of the study is represented in Figure 1.

## 3. Results

### 3.1. Sample Characteristics and Epidemiological Association with Bruxism

The studied population included 769 participants (59.7% females) with a mean age of 42.8 ± 18.8 years. As for the village of origin, the largest number of subjects were from Val di Resia (n = 274), while the smallest number were from Sauris (n = 76). The prevalence of bruxism in the population was 20.9% (161 affected individuals). Females showed a slightly higher prevalence of bruxism compared to males (21.4% versus 20.3%), but no significant association was found between bruxism and sex.

The characteristics of the studied cohort are reported In Table 1.

Among the possible risk factors, anxiety disorder showed a significant difference between cases and controls (Chi-Square test *p*-value = 0.01, Table 1), and this result was confirmed by logistic mixed effect models including the effect of age, sex, and village of origin (*p*-value = 0.015, Table 2). 

No associations were found for depressive disorders, smoking, and caffeine and alcohol consumption.

### 3.2. Genetic Associations with Bruxism and GTEx Data Analysis

**Figure 2:**
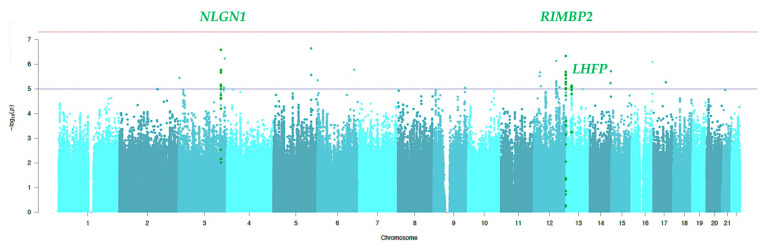
Manhattan plot for bruxism including 135 individuals affected by bruxism and 523 healthy subjects. The x-axis refers to the chromosomal position for each variant. The y-axis represents the −log10 *p*-value. The red horizontal line indicates the genome-wide significance threshold (*p*-value = 5 × 10^−8^), and the blue horizontal line indicates the suggestive significance threshold (*p*-value = 1 × 10^−5^). *NLGN1*: Neuroligin 1; *RIMBP2:* RIMS binding protein 2; *LHFP:* Lipoma HMGIC Fusion Partner.

Based on the mixed model results, GWAS was performed, adjusting for anxiety disorder as well as age, village, sex, and the first 10 principal components. Figure 2 shows the Manhattan plot of GWAS results performed on a cohort of 658 individuals (135 individuals presenting bruxism and 523 healthy individuals).

The GWAS analysis highlighted a total of 55 variants associated with bruxism (*p*-value <10^−5^) listed in Table S1.

Table 3 shows the most relevant results, selected according to the prioritization rules reported in Section 2.5 (i.e., only coding protein genes and at least five SNPs with a *p*-value < 10^−5^ in the locus).

**Figure 3:**
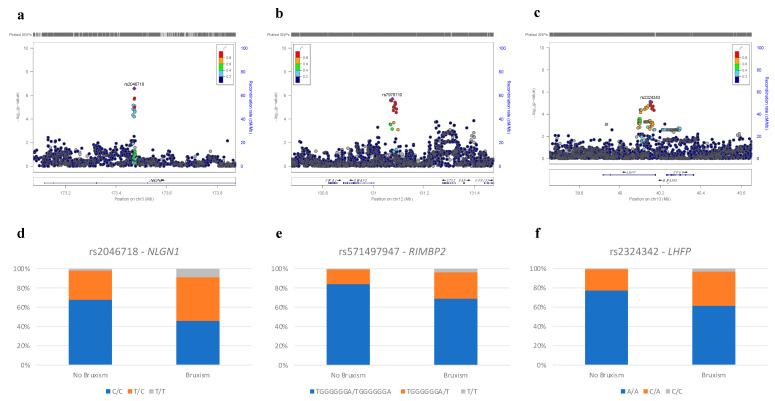
Regional plot for the identified *loci* (**a**–**c**) and Bar plot illustrating the percentage of individuals affected by bruxism for each genotype of the top SNPs of the three *loci* identified by the GWAS (**d**–**f**). Regional plot for the identified *loci* on chromosome 3 (**a**), 12 (**b**), and 13 (**c**). The top SNP, rs2046718 (**a**), rs2324342 (**c**), and the second SNP rs7978710 (**b**) of the region (since the top SNP of the region, rs571497947, was not available for the plot) are highlighted. The y–axis represents the −log10 of the *p*-values derived from the meta-analysis. Variants’ position is reported on the x–axis, alongside the *NLGN1* gene (**a**), *RIMBP2* gene (**b**), and *LHFP* gene (**c**).

GWAS results allowed the identification of three novel regions associated with bruxism. The first region was detected on chromosome 3, near the Neuroligin 1 (*NLGN1*) gene (Figure 3a); individuals carrying the C allele of the most associated SNP, namely rs2046718, had a greater risk of developing bruxism than those with the T allele (Table 3 and Figure 3d). The second region was identified on chromosome 12, near the RIMS Binding Protein 2 (*RIMBP2*) gene (Figure 3b); individuals carrying the T allele of the top SNP rs571497947 had a risk of bruxism 3.495 times higher than individuals with the TGGGGGGA allele sequence (Table 3 and Figure 3e). Lastly, the third region was identified on chromosome 13, near the Lipoma HMGIC Fusion Partner (*LHFP*) gene (Figure 3c); individuals with the C allele of the top SNP rs2324342 had a 2.752 times higher risk of bruxism compared to individuals with the A allele (Table 3 and Figure 3f).

**Figure 4:**
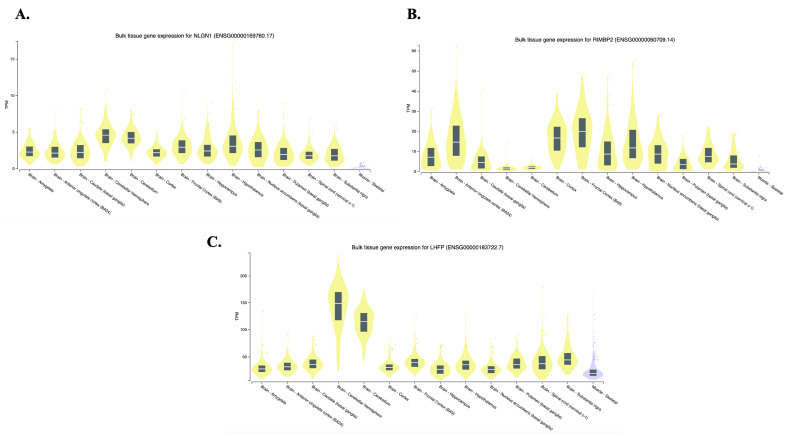
Tissue-specific expression (GTEx v8) of the identified genes in possible bruxism-related tissues (Brain, Skeletal muscle). (**A**) *NLGN1* gene expression, (**B**) *RIMBP2* gene expression, (**C**) *LHFP* gene expression. TMP = transcripts per million. These data were downloaded from the GTEx Portal (https://www.gtexportal.org/home/, accessed on 5 July 2023).

Expression levels of the identified genes were checked into the GTEx database, as shown in Figure 4. Overall, all three analyzed genes show moderate expression in brain tissues.

In particular, the *NLGN1* gene displays a slightly higher expression in the hypothalamus, *RIMBP2* gene in the frontal cortex, and *LHFP* in the cerebellar hemisphere tissue.

## 4. Discussion

Bruxism is a common complex condition involving involuntary teeth grinding and clenching, with an overall prevalence ranging from 8% to 31.4% [3,4,5,6,7]. 

To date, several environmental risk factors have been identified, such as smoking, alcohol and caffeine consumption, sleep disturbances, medications, and drug intake [10,12,13]. Further, a growing body of literature has highlighted the possible involvement of several psychosocial factors, including anxiety, stress, and mood disorders [10,14,15,16]. 

Several theories have been proposed to explain its etiopathogenesis; however, the majority of studies agree that it is a CNS-mediated phenomenon [37].

However, the comprehension of the precise environmental and molecular mechanisms underlying bruxism onset is still far from complete.

In order to dissect the complex etiology of multifactorial conditions, genetically isolated populations have been proven to be the ideal study sample since they are characterized by high environmental and genetic homogeneity that facilitates the identification of etiological factors. 

In this light, this study aims to investigate environmental risk factors and genes and variants underlying bruxism etiopathogenesis, taking advantage of accurately characterized Italian genetically isolated populations, the FVG cohort, in the framework of the “FVG Genetic Park” project [29].

In this study population (n = 769), the prevalence of bruxism was 20.9%, in accordance with the overall prevalence of bruxism reported in the literature [3,4,5,6,7,8].

Regarding the first aim of our study, in order to identify possible risk factors for bruxism development, epidemiological analyses were carried out. Specifically, a statistically significant association between bruxism and anxiety was detected, confirming previous literature data [10,14,15,16,38]. Indeed, a recent systematic review and meta-analysis of the literature reported that bruxism is triggered by alterations of neural pathways and catecholamines (e.g., dopamine) neurotransmission that controls masticatory muscles [39]. Abnormal fluctuations in dopamine levels in the brain are often associated with chronic stress; notably, individuals with anxiety disorders tend to manifest their emotional pressure with bruxism [39]. Therefore, it has been hypothesized that emotional distress can cause the activation of the sympathetic nervous system, leading to the release of catecholamines, especially dopamine, that could cause repetitive jaw-muscle activity, typical of bruxism [21,22]. Indeed, higher levels of catecholamines have been detected in the urine of individuals presenting with bruxism compared to controls [15,40,41]. In this regard, bruxism might be considered as a “psychic stress valve” for the mitigation of anxiety. 

Studies in the literature state a positive association between bruxism and smoking, caffeine, and alcohol consumption [10,12,13], but in this study no significant associations were identified. However, it has to be taken into account that these results might be influenced by several factors, including the difficulty of establishing a uniform threshold level of consumption of these substances, as well as the criteria employed to diagnose bruxism in the various studies performed [10].

Overall, the statistical results of the present study give support to the theory that describes bruxism as a CNS-mediated phenomenon, thus highlighting the fundamental role of brain-related mechanisms underlying its etiopathogenesis.

Regarding genetics, GWAS is a successful strategy to point out novel candidate genes potentially involved in multifactorial conditions.

Taking advantage of this approach, in this study, three novel candidate genes, i.e., *NLGN1*, *RIMBP2*, and *LHFP*, were identified as potential risk loci for bruxism. 

Of note, all of the three genes are expressed in the brain [36], further supporting the critical role of the CNS in relation to bruxism.

In detail, the *NLGN1* gene encodes a neuronal transmembrane protein, Neuroligin 1, specifically localized to the postsynaptic compartment. The *NLGN1* gene modulates the formation and maturation of central nervous system synapses in the mammalian brain [42,43], thus regulating glutamatergic excitatory synapse remodeling [44], and contributing to the regulation of proper excitatory and/or inhibitory impulses in determinate neural connections [42,43,44,45]. Studies in the literature have reported that Neuroligin-1 is required for associative fear and spatial memory storage [46,47]. Indeed, *Nlgn1* knock-out mice exhibit behavioral changes resulting in impaired spatial memory and increased repetitive behaviors, which are linked to cerebellar and orbitofrontal cortex abnormalities [48].

Variants within the *NLGN1* gene and alterations of the Neuroligin 1 synaptic pathway are reported to be involved in the etiopathogenesis of autism, neurodevelopmental disorders [49,50], memory loss, and depression [51,52], thus highlighting this gene’s relevance in brain function.

Notably, a GWAS published in 2016 supported the potential involvement of the *NLGN1* gene in post-traumatic stress disorder onset [53]. According to the findings, carriers of *NLGN1* variants exhibit increased activation of the limbic and prefrontal regions and connections between the amygdala and the dorsal–lateral prefrontal cortex. This evidence indicates *NLGN1* as a probable regulator of the neural connection of the hippocampus-amygdala–prefrontal axis [53]. Considering that also bruxism could be caused by an alteration of the hippocampus-amygdala–hippocampus-prefrontal circuit [54], it can be speculated that variants within *NLGN1* could be responsible for determining bruxism onset. This is the first study that associates this gene with this condition, and further functional studies with in vivo models will be essential to validate this finding.

The *RIMBP2* (Rab-Interacting Molecule-Binding Protein 2) gene encodes a multidomain protein known to regulate pre-synaptic transmission [55]. 

In particular, it is considered a central organizer of voltage-gated Ca^2+^ channel disposition at the synaptic cleft and synaptic vesicle release sites, promoting their tethering and docking [56].

Indeed, studies in animal models showed that *RIMBP2* gene deletion reduces the voltage-gated Ca^2+^ channel abundance and clustering at active zones (which are specialized regions at the presynaptic terminals where neurotransmitter release occurs), thus impairing synaptic vesicle tethering and docking [56,57,58,59]. Specifically, it was reported that the *RIMBP2* gene is critical to promote neurotransmitter release by regulating vesicle docking at hippocampal mossy fiber synapses [60]. Interestingly, alteration of hippocampal mossy fiber function has been described in relation to neuropsychiatric disorders onset [61], and mice models lacking mossy fiber cells displayed an anxiety-like behavior [62].

To further support this evidence, other studies in literature pointed out the possible involvement of *RIMBP2* in the development of behavioral abnormalities, such as bipolar disorders [63]; further, members of the RIMBP2 protein family have been described in relation to autism spectrum disorder onset [59]. This is the first time that this gene has been linked with bruxism; however, considering its biological function and its possible implication in anxiety and behavioral abnormalities development, it could be considered a promising candidate to elucidate the molecular mechanisms underlying bruxism.

Finally, the *LHFP* gene (also known as the *LHFPL6* gene) encodes a member of the lipoma HMGIC fusion partner protein, which is a tetraspan transmembrane protein first identified in lipomas [64].

*LHFP* is expressed in several tissues, including the brain; however, its function is not completely clear. Variants within the *LHFP* gene were described in relation to hippocampal volume regulation [65]. As reported previously, containing high levels of glucocorticoid receptors, the hippocampus is a key regulator of stress and anxiety behaviors, including emotion-induced bruxism [66,67]. To date, no studies in the literature have described an association between hippocampal volume and bruxism development; however, considering that altered hippocampal activity and volume have also been described in individuals suffering from anxiety, it could be possible to consider *LHFP* as a novel player underlying bruxism onset. Further functional studies will be needed to clarify this hypothesis [54].

This study presents limitations that need to be considered. First of all, this is an epidemiological retrospective cross-sectional and GWA study of data collected in 2008, so to develop a better model to deepen the knowledge of the studied phenotype, prospective cohort studies would be needed. In addition, to validate the obtained findings, a replica in other cohorts, such as other Italian-isolated populations and populations of other ethnic groups, is required.

In summary, this study allowed the identification of three new candidate genes, all expressed in brain tissues and potentially involved in the neurobiological mechanisms underlying bruxism etiopathogenesis. However, to date, there are no studies available in the literature that directly correlate variations in these genes to bruxism development, and therefore, the biological mechanisms underlying these associations need to be fully characterized.

## 5. Conclusions

This study takes advantage of an accurately clinically characterized cohort of Italian genetically isolated populations, with the final goal of shedding light on the complex mechanisms underlying bruxism.

The detailed clinical odontostomatological evaluation and the assessment of bruxism by expert dentists, combined with the homogeneous environmental background of this cohort, allowed to confirm anxiety as a risk factor for bruxism, in line with previous reports.

Regarding genetics, GWAS analysis led to the identification of three promising new candidate genes, namely *NLGN1*, *RIMBP2*, and *LHFP* genes. Considering their neurobiological roles, these genes could be considered as novel molecular players underlying bruxism onset and etiopathogenesis. 

Overall, these findings significantly support the CNS-mediated theory of bruxism, thus opening novel perspectives on the molecular mechanisms at the base of this condition onset.

Further functional studies will be essential for validating these results; in particular, replication of the obtained results in independent cohorts and functional studies with in vitro and in vivo models will be needed to deepen the relevance and molecular roles of the identified genes in relation to bruxism development. 

Indeed, a deeper understanding of bruxism etiopathogenesis would be essential to developing novel treatment approaches and strategies with the ultimate goal of improving affected individuals’ quality of life.

## Figures and Tables

**Table 1:** Characteristics of participants in all samples and according to bruxism presence (cases)/absence (controls). Numbers and percentages in brackets were employed for data description except for age (mean ± standard deviation), number of cigarettes/day, number of cups/day, and grams of alcohol/day (median (IQR)). The *p*-value is referred to *t*-test, Mann–Whitney U test, or Chi-Square test to evaluate the difference in the distribution of the parameters between cases and controls. NS: not significant; SD: standard deviation; IQR: interquartile range. ***^#^*** Available for 699 individuals, 152 with bruxism and 547 without. ***^##^*** Available for 687 individuals, 148 with bruxism, and 539 without. ***^###^*** Available for 687 individuals, 149 with bruxism and 538 without. * Available for 128 individuals, 27 with bruxism and 101 without. ** Available for 566 individuals, 126 with bruxism and 440 without. *** Available for 420 individuals, 97 with bruxism and 323 without.

Parameter		All (n = 769)	Cases (n = 161)	Controls (n = 608)	*p*-Value
Age (mean ± SD)		42.8 ± 18.81	45.78 ± 17.79	42.02 ± 19.01	0.0195
Sex	Female	459 (59.7)	98 (60.9)	361 (59.4)	NS
	Male	310 (40.3)	63 (39.1)	247 (40.6)	
Village of origin	Clauzetto	146 (19.0)	53 (32.9)	93 (15.3)	<0.001
	Erto-Casso	119 (15.5)	28 (17.4)	91 (15.0)	
	Illegio	154 (20.0)	22 (13.7)	132 (21.7)	
	Val di Resia	274 (35.6)	49 (30.4)	225 (37.0)	
	Sauris	76 (9.9)	9 (5.6)	67 (11.0)	
Level of education	Elementary	168 (22.6)	34 (21.5)	134 (22.9)	NS
	Lower secondary	238 (32.1)	52 (32.9)	186 (31.8)	
	Upper secondary	274 (36.9)	60 (38.0)	214 (36.6)	
	University	62 (8.4)	12 (7.6)	50 (8.6)	
Anxiety disorder		32 (4.2)	13 (8.1)	19 (3.1)	0.0100
Depressive disorder		49 (6.4)	10 (6.2)	39 (6.4)	NS
Smoke ^#^	Smokers	131 (18.7)	28 (18.4)	103 (18.8)	NS
	Number of cigarettes/day * median (IQR)	10 (5–20)	12 (9–20)	10 (5–15)	NS
Caffeine consumption ^##^	Consumers	579 (84.3)	128 (85.9)	451 (83.8)	NS
	Number of cups/day ** median (IQR)	2 (1.6–3.5)	2 (2–3.5)	2 (1.5–3.5)	NS
Alcohol consumption ^###^	Consumers	429 (62.4)	100 (67.6)	329 (61.0)	NS
	Grams of alcohol/day *** median (IQR)	13.5 (2.7–27.8)	13.5 (5.2–28.4)	13 (2.6–27.6)	NS

**Table 2:** Results of the logistic mixed model employed to verify the impact of anxiety on bruxism. The model was adjusted for the following parameters: age (fixed effects), sex (fixed effects), and village of origin (random effect). OR: Odds Ratios CI: Confidence Intervals, M: male.

Predictor	OR	95% CI	*p*-Value
Sex, M	0.96	0.66–1.38	0.818
Age	1.01	1.00–1.02	0.093
Anxiety disorder	2.54	1.20–5.38	0.015

**Table 3:** Results of GWAS on bruxism: new candidate genes. Chr: chromosome; Position: position of the most associated SNP in base pair (genetic data were aligned to the Human genome reference build 37 (GRCh37)); Alleles: other allele/risk allele; Freq: frequency of the risk allele; OR: Odds Ratio; 95% CI: 95% Confidence Interval; N SNPs: Number of SNPs with a *p*-value < 1 × 10^−^^5^ in each locus.

Top SNP	Nearest Genes	Chr	Position	Alleles	Freq	OR	95% CI	*p*-Value	N SNPs
rs2046718	*NLGN1*	3	173472327	C/T	0.200	2.649	1.828–3.838	2.63 × 10^−7^	6
rs571497947	*RIMBP2*	12	131072229	TGGGGGGA/T	0.101	3.495	2.148–5.685	4.68 × 10^−7^	14
rs2324342	*LHFP*	13	40150635	A/C	0.136	2.752	1.767–4.286	7.47 × 10^−6^	6

## Data Availability

Data are already available in the European Genomephenome Archive (EGA) at the following links. BAM files https://www.ebi.ac.uk/ega/studies/EGAS00001000252 (accessed on 5 July 2023); sample list, vcf files https://www.ebi.ac.uk/ega/studies/EGAS00001001597 (accessed on 5 July 2023); https://www.ebi.ac.uk/ega/datasets/EGAD00001002729 (accessed on 5 July 2023).

## Supplementary Materials

The following supporting information can be downloaded at: https://www.mdpi.com/article/10.3390/biomedicines12020304/s1, Table S1. All suggestive results of GWAS (*p*-value < 10^−5^).
