# Supplementary material for: Clenching the Strings of Bruxism Etiopathogenesis: Association Analyses on Genetics and Environmental Risk Factors in a Deeply Characterized Italian Cohort
Source: Biomedicines. 2024 Jan 28;12(2):304. doi: 10.3390/biomedicines12020304 (PMC10887134; doi:10.3390/biomedicines12020304)
Supplement: Supplementary file 1 [file biomedicines-12-00304-s001.zip › Table S1.pdf]

Table S1. All suggestive results of GWAS (*p*-value<10^{-5}). Chr = chromosome; Position = position of the best SNP in base pair (all data are aligned to the Human genome reference build 37 (GRCh37)); Alleles = other allele/risk allele; Freq = frequency of the risk allele; OR = Odds Ratio; 95% CI = 95% Confidence Interval; N SNPs: Number of SNPs with a p-value < $1x10^{-5}$.

Chr	*Position*	*SNP*	*Alleles*	*Freq*	*OR (95% CI)*	*p-value*	*Consequence*	*Gene*	*Biotype*
3	3500995	rs201276817	T/C	0.064	5.32 (2.62 - 10.78)	3.6E-06	intron_variant	*AC026188.1*	lincRNA
3	173468188	rs1038644	A/G	0.105	3.13 (1.90 - 5.14)	6.8E-06	intron_variant	*NLGN1*	protein_coding
3	173472086	rs4465969	A/G	0.215	2.46 (1.69 - 3.56)	2.1E-06	intron_variant	*NLGN1*	protein_coding
3	173472327	rs2046718	C/T	0.200	2.65 (1.83 - 3.84)	2.6E-07	intron_variant	*NLGN1*	protein_coding
3	173473286	rs1387003	A/T	0.215	2.48 (1.71 - 3.59)	1.7E-06	intron_variant	*NLGN1*	protein_coding
3	173473923	rs147792696	T/TACACAA	0.226	2.35 (1.61 - 3.42)	8.1E-06	intron_variant	*NLGN1*	protein_coding
3	173474556	rs397774661	T/TA	0.216	2.53 (1.72 - 3.72)	2.2E-06	intron_variant	*NLGN1*	protein_coding
3	185155430	rs5855065	C/CTT	0.127	2.85 (1.79 - 4.51)	9.0E-06	intron_variant	*MAP3K13*	protein_coding
3	189128814	rs112271984	T/C	0.065	4.38 (2.45 - 7.83)	6.0E-07	intergenic_variant	-	-
5	154794656	rs397945106	A/AT	0.214	2.77 (1.88 - 4.07)	2.3E-07	intergenic_variant	-	-
5	154797478	rs59842935	A/C	0.197	2.55 (1.73 - 3.78)	2.7E-06	intergenic_variant	-	-
6	1405632	rs9378620	A/T	0.078	4.24 (2.29 - 7.87)	4.5E-06	intergenic_variant	-	-
6	150829462	rs530877947	C/CTT	0.154	3.30 (2.02 - 5.38)	1.7E-06	intron_variant	*RP11-291C6.1*	processed_pseudogene
9	129053048	rs35610872	G/A	0.566	1.93 (1.44 - 2.59)	9.2E-06	intergenic_variant	-	-
12	23752325	rs79109875	A/G	0.068	4.42 (2.37 - 8.24)	3.1E-06	intron_variant	*SOX5*	protein_coding
12	25195936	rs397790236	T/TA	0.809	2.93 (1.88 - 4.57)	2.2E-06	downstream_gene_variant	*LRMP*	processed_transcript
12	29032311	rs767855632	G/GAAAA	0.105	3.82 (2.12 - 6.88)	7.9E-06	intergenic_variant	-	-
12	92071049	rs10859178	G/A	0.198	2.40 (1.64 - 3.53)	7.6E-06	upstream_gene_variant	*RP11-1041F24.1*	lincRNA
12	92072728	rs10859179	A/G	0.199	2.40 (1.64 - 3.52)	7.6E-06	upstream_gene_variant	*RP11-1041F24.1*	lincRNA
12	92079915	rs1493762	T/G	0.197	2.43 (1.66 - 3.55)	5.1E-06	regulatory_region_variant	-	enhancer
12	92080205	rs1493761	A/G	0.197	2.43 (1.66 - 3.56)	5.1E-06	intergenic_variant	-	-
12	92084642	rs9634202	C/T	0.197	2.43 (1.66 - 3.57)	5.0E-06	intergenic_variant	-	-
12	92089340	rs75841027	AATG/A	0.197	2.42 (1.65 - 3.55)	5.8E-06	intergenic_variant	-	-
12	92090271	rs10859181	A/G	0.197	2.41 (1.65 - 3.54)	6.0E-06	intergenic_variant	-	-
12	92092330	rs10859182	G/A	0.197	2.41 (1.64 - 3.53)	6.5E-06	intergenic_variant	-	-
12	92099539	rs369634297	TAGAAGC/T	0.196	2.43 (1.66 - 3.57)	6.0E-06	intergenic_variant	-	-

12	92102903	rs11106253	T/C	0.166	2.78 (1.85 - 4.16)	7.4E-07	intergenic_variant	-	-
12	92108761	rs116634769	T/G	0.197	2.41 (1.64 - 3.53)	6.4E-06	intergenic_variant	-	-
12	92109755	rs11323014	CT/C	0.197	2.41 (1.64 - 3.53)	6.4E-06	intergenic_variant	-	-
12	92113658	rs28489833	G/A	0.166	2.70 (1.75 - 4.17)	7.6E-06	intergenic_variant	-	-
12	105155242	12:105155242_TACACACAC_TAC	TACACAC/T	0.486	2.17 (1.54 - 3.05)	8.5E-06	3_prime_UTR_variant	*CHST11*	protein_coding
12	131068531	rs73456741	C/T	0.095	3.27 (1.99 - 5.35)	2.6E-06	intron_variant	*RIMBP2*	protein_coding
12	131069632	rs57326734	G/A	0.096	3.26 (1.99 - 5.34)	2.7E-06	intron_variant	*RIMBP2*	protein_coding
12	131069730	rs12315215	C/T	0.095	3.26 (1.99 - 5.33)	2.8E-06	intron_variant	*RIMBP2*	protein_coding
12	131069907	rs11061011	G/A	0.095	3.26 (1.99 - 5.34)	2.8E-06	intron_variant	*RIMBP2*	protein_coding
12	131072117	rs369673290	C/T	0.096	3.25 (1.98 - 5.32)	2.8E-06	intron_variant	*RIMBP2*	protein_coding
12	131072229	rs571497947	TGGGGGGA/T	0.101	3.49 (2.15 - 5.69)	4.7E-07	intron_variant	*RIMBP2*	protein_coding
12	131075463	rs7978710	G/A	0.095	3.32 (2.02 - 5.45)	2.1E-06	intron_variant	*RIMBP2*	protein_coding
12	131080871	rs7968068	T/G	0.109	2.93 (1.82 - 4.71)	9.5E-06	intron_variant	*RIMBP2*	protein_coding
12	131085414	rs58481716	C/T	0.108	2.97 (1.85 - 4.76)	6.6E-06	intron_variant	*RIMBP2*	protein_coding
12	131086167	rs12313276	G/A	0.108	3.05 (1.90 - 4.90)	3.8E-06	intron_variant	*RIMBP2*	protein_coding
12	131087275	rs7974202	G/A	0.108	3.00 (1.87 - 4.82)	5.3E-06	intron_variant	*RIMBP2*	protein_coding
12	131087471	rs11061017	C/T	0.108	3.00 (1.87 - 4.82)	5.4E-06	intron_variant	*RIMBP2*	protein_coding
12	131088062	rs728931	G/A	0.108	3.00 (1.87 - 4.82)	5.3E-06	intron_variant	*RIMBP2*	protein_coding
12	131088248	rs7972304	A/T	0.108	3.00 (1.87 - 4.82)	5.3E-06	intron_variant	*RIMBP2*	protein_coding
13	40150635	rs2324342	A/C	0.136	2.75 (1.77 - 4.29)	7.5E-06	intron_variant	*LHFP*	protein_coding
13	40151066	rs2324343	G/T	0.136	2.75 (1.77 - 4.28)	7.5E-06	intron_variant	*LHFP*	protein_coding
13	40152431	rs4142372	G/A	0.137	2.73 (1.75 - 4.24)	8.8E-06	intron_variant	*LHFP*	protein_coding
13	40152988	rs9315709	C/A	0.137	2.72 (1.75 - 4.24)	8.9E-06	intron_variant	*LHFP*	protein_coding
13	40154473	rs9576869	T/C	0.137	2.72 (1.75 - 4.23)	8.4E-06	intron_variant	*LHFP*	protein_coding
13	40156826	rs2324345	C/T	0.131	2.76 (1.77 - 4.31)	7.9E-06	intron_variant	*LHFP*	protein_coding
14	103838633	14:103838633_G_GCA	G/GCA	0.715	2.64 (1.73 - 4.01)	5.9E-06	downstream_gene_variant	*RP11-600F24.1*	processed_pseudogene
14	105107537	rs7143644	G/A	0.071	4.75 (2.50 - 9.03)	1.9E-06	intergenic_variant	-	-
16	86064942	rs868325	C/T	0.240	2.52 (1.74 - 3.64)	8.3E-07	regulatory_region_variant	-	open_chromatin_region
17	50853054	17:50853054_TAAAAAAAAAAAAA_T	TAAAAAAAAAAAAA/T	0.093	4.29 (2.29 - 8.05)	5.5E-06	intergenic_variant	-	-
